# A time-domain view of charge carriers in semiconductor nanocrystal solids

**DOI:** 10.1039/c9sc05925c

**Published:** 2020-05-07

**Authors:** Wenbi Shcherbakov-Wu, William A. Tisdale

**Affiliations:** Department of Chemistry, Massachusetts Institute of Technology Cambridge MA 02139 USA; Department of Chemical Engineering, Massachusetts Institute of Technology Cambridge MA 02139 USA tisdale@mit.edu

## Abstract

The movement of charge carriers within semiconductor nanocrystal solids is fundamental to the operation of nanocrystal devices, including solar cells, LEDs, lasers, photodetectors, and thermoelectric modules. In this perspective, we explain how recent advances in the measurement and simulation of charge carrier dynamics in nanocrystal solids have led to a more complete picture of mesoscale interactions. Specifically, we show how time-resolved optical spectroscopy and transient photocurrent techniques can be used to track both equilibrium and non-equilibrium dynamics in nanocrystal solids. We discuss the central role of energetic disorder, the impact of trap states, and how these critical parameters are influenced by chemical modification of the nanocrystal surface. Finally, we close with a forward-looking assessment of emerging nanocrystal systems, including anisotropic nanocrystals, such as nanoplatelets, and colloidal lead halide perovskites.

## Abstract 2

Time-domain spectroscopy and transient photocurrent techniques have revealed new understanding of mesoscale carrier dynamics in nanocrystal solids, including the role of energetic disorder, interactions with trap states, and nonequilibrium dynamics

## Graphical abstract

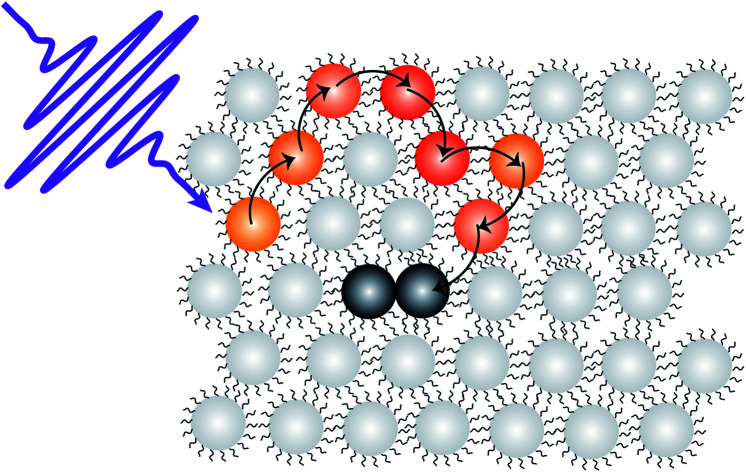

## Introduction

Colloidal semiconductor nanocrystals (NCs), or quantum dots (QDs), have attracted considerable attention over the past three decades due to a wide range of potential technological applications, including light-emitting diodes (LEDs), solar cells, photodetectors, and biological imaging.^1–6^ As the semiconductor size is reduced to the nanometer length scale, quantum confinement restricts the allowed momentum of charge carriers within each NC, leading to discretization of the electronic energy levels and an increase in the effective bandgap.^7–9^ Excitons, coulombically bound electron–hole pairs, form upon photoexcitation in NCs. The spatial extent of the exciton, often parameterized by the exciton Bohr radius, provides a convenient estimate of the length scale below which NCs start to exhibit quantum size effects. When the NC size is significantly smaller than the exciton Bohr radius, the NC is labeled as “strongly quantum confined”.^10^

In this perspective, we focus primarily on nominally-spherical NCs that are strongly quantum confined, such as cadmium and lead chalcogenide quantum dots. We begin with a brief review of electronic coupling in nanocrystal solids and discuss different theoretical frameworks for describing electron transfer between NCs. We then describe recent developments in time-domain measurement of charge carriers in NC solids and contrast those with more traditional steady-state techniques, summarizing both the advantages and disadvantages. The following sections discuss new insights revealed by time-domain techniques, including behavior of trap states and multicarrier interactions. We close with a discussion of new classes of semiconductor nanocrystals that may exhibit behavior different from those studied so far: (i) anisotropic NCs, such as atomically-thin nanoplatelets that are very strongly quantum confined, and (ii) lead halide perovskite NCs.

## Action at the mesoscale

Significant attention in the colloidal nanocrystal field has been directed toward the single-particle level, such as electronic structure of the individual NCs,^11,12^ or on the device level, such as the performance of solar cells and LEDs.^4,5^ Intermediate between these regimes is the mesoscale, wherein the interaction between small numbers of discrete NCs dictates charge carrier behavior within NC solids.^13–17^ Highly efficient devices require maximal packing of NCs in their active layers, bringing NCs into close electrical contact. The optical and electronic properties of NC solids with strong electronic coupling are often different from those of the isolated NCs. A complete understanding of mesoscale dynamics in NC solids is central to the understanding of charge mobility and lifetime, and ultimately, device efficiency. In the following paragraphs, we will review the basics of interparticle charge transport and the recent advances in the field.

**Fig. 1:**
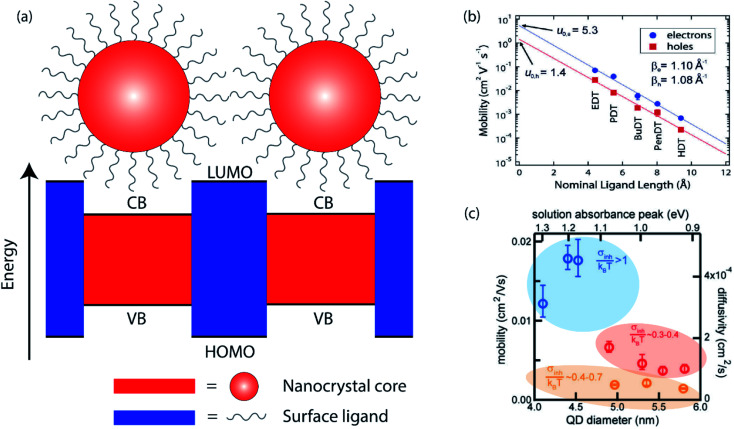
(a) Cartoon illustrating the “particle-in-a-sphere” model of two neighboring nanocrystals. (b) Carrier mobility as a function of ligand length in ambipolar PbSe NC field-effect transistors (reprinted with permission from ref. 18. Copyright 2010 American Chemical Society). (c) Calculated intrinsic charge carrier mobility and diffusivity as a function of PbS NC size (reprinted with permission from ref. 22. Copyright 2017 American Chemical Society).

In the simplest approximation, the core electronic states of a nanocrystal are described by the “particle-in-a-sphere” model, and these states are spatially separated by the surface ligands which act as potential barriers between NCs, as shown in Fig. 1a. The WKB approximation offers a convenient estimate of the charge carrier transmission probability (*T*) through this potential barrier,1
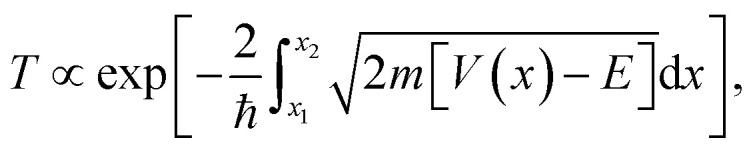
where *m* is the mass of the particle and *V*(*x*) − *E* is the effective barrier height. As can be seen in the mathematical form, the tunneling probability decreases exponentially with barrier width and the square root of height. Additionally, the particle with higher kinetic energy, *E*, has a greater chance of tunneling through the barrier. While atomistic simulations provide a more accurate picture of electronic structure of nanocrystals, eqn (1) can give some intuition into how varying physical parameters, such as NC size, can influence the charge carrier tunneling.

The electronic coupling in NC solids is highly tunable, as it is determined by a number of controllable factors. The potential barrier width is directly related to the surface ligand length. It has been shown that in PbSe NC solids treated with a series of alkanedithiol with different alkane lengths, the carrier mobilities, both electrons and holes, decrease exponentially with increasing ligand length, shown in Fig. 1b.^18^ Mobility studies, such as field effect transistor (FET) studies, reveal a tunneling decay parameter similar to that measured for charge transfer in donor–acceptor junction systems.^19^ Ligands contribute to the electronic structure not only through the length, but also through the chemical nature of the anchor groups. Even though the electron affinities of the commonly utilized ligands, such as alkyldiamines, alkyldithiols and alkyldicarboxylic acids, are not expected to vary much, different charge carrier mobilities have been observed in NC solids treated with different ligands, leading to the hypothesis that the different functional groups result in different potential barrier heights.^20^ Another contributing factor to the electronic structure is the NC size, seen in Fig. 1c. It is well-known that different NC sizes lead to different energy bandgaps, shifting the relative positions of conduction band (CB) of the NC and lowest unoccupied molecular orbitals (LUMO) of the surface ligands. More importantly, the kinetic energy of a carrier also changes as a function of NC sizes, altering the effective energy barrier between NCs.^21–23^ Lastly, the NC shape, composition, and NC assembly structure can all have an effect on the electronic coupling between neighboring nanocrystals by changing the electronic structure.

Site-to-site hopping remains the dominant charge transport mechanism in most NC solids. In this incoherent regime, the charge carrier (electron or hole) spends most of its time localized on an individual nanocrystal, but undergoes periodic stochastic “jumps” from NC to NC. In the simplest approximation, the probability per unit time of making a transition from NC *i* to NC *j* can be described by the Miller–Abrahams rate equations:2
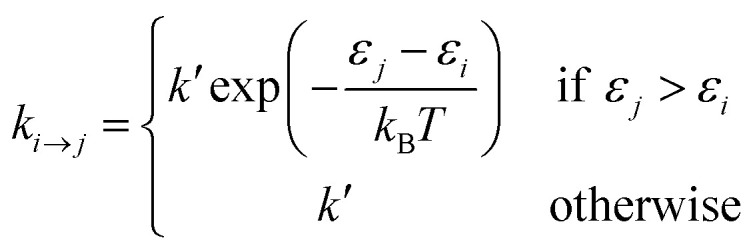
where *ε*_*i*_ and *ε*_*j*_ are the energies of transport levels of NCs *i* and *j*, *k*_B_*T* is the product of Boltzmann's constant and temperature, and *k*′ = *k*_0_ exp(−*βr*_*ij*_). Here *k*_0_ is the attempt frequency, *β* is the tunneling decay constant and *r*_*ij*_ is the edge-to-edge distance between the two NCs. Intuitively, this set of equations shows that energetically uphill hops are slower than downhill hops.

A more complete description of localized hopping is provided by Marcus theory.^19,24,25^ In the Marcus picture, the electron transfer event itself is assumed to be fast relative to the timescale of fluctuations in the dielectric environment, and the rate-limiting step is the time it takes for the donor NC, acceptor NC, and their environment to stochastically achieve a transition state configuration in which it is isoenergetic for the charge to be found either on the donor or the acceptor NC. Both the Marcus rate expression and the Miller–Abrahams expression (eqn (2)) describe thermally-activated charge transfer processes between localized sites. However, Marcus theory also predicts variation in the rate of energetically downhill charge transfer events. Though Marcus theory has successfully described experimental observations in some NC systems,^26–29^ we have generally preferred to use the Miller–Abrahams expression in our work because (1) the reorganization energy is expected to be small in most NC solids,^30^ where no polar solvent is present, and (2) eqn (2) has fewer fitting parameters than the equivalent Marcus rate expression.

Recent developments in colloidal synthesis and ligand exchange have enabled significant progress in the formation of NC superlattices.^31^ In these highly ordered NC solids, charge carriers may delocalize over several NC sites, and coherent transport may become possible.^16,32–35^ However, the mechanisms of charge transport in the strong coupling regime are still debated.^17,36^

## Steady-state *vs.* dynamic measurements of charge transport

**Fig. 2:**
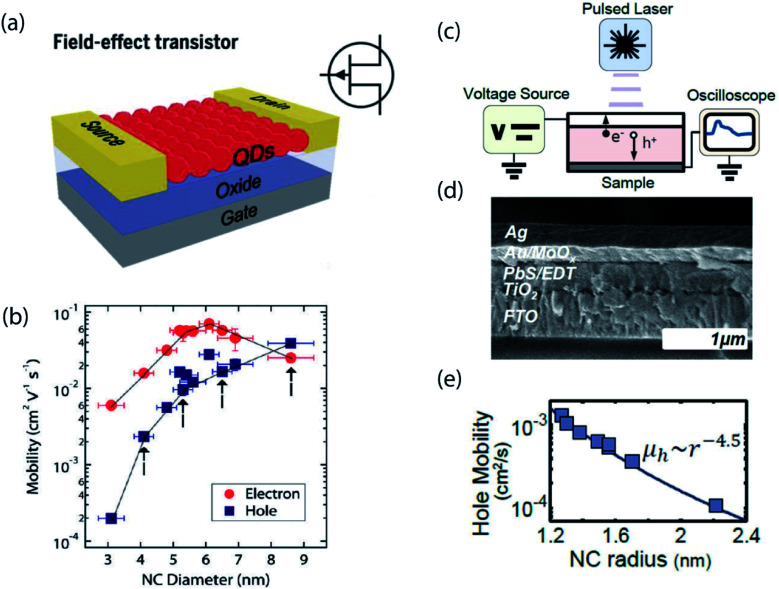
(a) Schematic of a typical field effect transistor (reproduced from ref. 2 with permission by AAAS). (b) Carrier mobility as a function of nanocrystal size in ambipolar PbSe NC field-effect transistors (reprinted with permission from ref. 18. Copyright 2010 American Chemical Society). (c) Schematic of a time-of-flight measurement set-up. (d) Cross-section SEM image of the device used in the TOF measurement. (e) Hole mobility as a function of nanocrystal size measured in the TOF measurement (reprinted with permission from ref. 21. Copyright 2014 American Chemical Society).

One of the first widely utilized methods for studying carrier transport in NC solids was *via* fabrication of field-effect transistors (FETs).^18,37,38^ In these experiments, multiple layers of NCs are deposited onto doped silicon substrates coated with a thermal SiO_2_ gate oxide, as illustrated in Fig. 2a. On the top, source and drain electrodes are patterned on each side of the NC film, creating a lateral device architecture, and the source–drain current is measured as a function of applied gate voltage. The electron and hole mobilities, *μ*, are usually calculated using the gradual channel approximation equation in the linear regime, which takes into account factors such as channel width, channel length and capacitance. While the FET mobility is certainly the most relevant metric for switching speeds in electrical circuits, it may not be the purest metric for probing mesoscale physics. For example, Wood and co-workers pointed out that the FET mobility is an *effective* mobility – including contributions from both trapped and free carriers – rather than the intrinsic mobility of carriers at the band edge.^21^

Alternatively, there exist dynamic electrical measurements. One example is the time-of-flight (TOF) photocurrent measurement, as shown in Fig. 2c and d.^21^ In this experiment, the material of interest lies between two contacts which are fixed at a given potential, *V*_0_. A short laser pulse is then used to generate a charge distribution in the material at one of the contacts, and the resulting displacement current transient is measured. The transient nature of the measurement enables isolation of the free hole mobility, in contrast to the effective carrier mobility obtained by FET.

The difference between steady-state and time-dependent measurements can be illustrated through the effect of NC size on carrier mobility and conductivity, with steady-state and dynamic measurements often revealing opposite trends. In an FET measurement of PbSe NCs, the hole mobility was observed to monotonically increase with increasing NC size, shown in Fig. 2b, whereas time-of-flight measurements revealed the opposite trend of decreasing mobility with increasing size, shown in Fig. 2e. Both experiments contain useful information, but care should be taken when interpreting the data.

Time-domain optical spectroscopy has emerged as an alternative method to probe mesoscale dynamics in NC solids.^22,39–46^ Several advantages of using an optical probe rather than electrical current are: (1) eliminating contact effects; (2) sensitivity to the whole film – rather than the layer closest to the gate dielectric; (3) ability to capture early-time non-equilibrium phases of transport; and (4) ability to spectrally distinguish trapped carriers from carriers at the band edge. Our group has performed ps–ns transient absorption (TA) experiments on different sized PbS NCs and extracted carrier mobility trends consistent with that from the TOF photocurrent measurement, as seen in Fig. 1c.^22^ In addition to the TA approach, transient photoluminescence and terahertz experiments can also provide time-domain charge carrier dynamics information,^47,48^ as can time-resolved microscopy approaches.^40,42,49^

However, there are notable limitations to such time-domain optical spectroscopy methods. Disadvantages include: (1) all-optical methods have difficulty distinguishing between exciton- and charge-transport, or identifying the sign of the dominant charge carrier (*i.e.* electron *vs.* hole transport); (2) in general, the sensitivity of electrical current measurements is much greater than absorption-based optical measurements; (3) care must be taken in extrapolating the implications of time-domain optical spectroscopy results for devices that operate at steady state; however, experiments can often be designed in a such a way that the perturbation is minimal. Finally, we emphasize that *both* approaches – electrical and optical – require a model to connect the macroscopic observables to microscopic insight.

## Early time carrier transport is dominated by energetic disorder

**Fig. 3:**
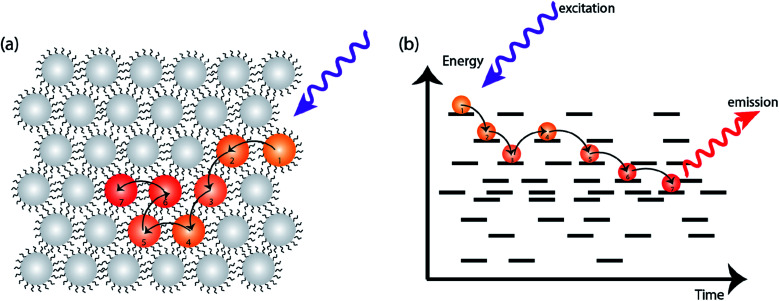
(a) Schematic for an energetically disordered NC ensemble. When excited by a high-energy light pulse, charge carriers migrate toward lower-energy sites within the ensemble. (b) The average energy of charge carriers decreases over time due to thermalization with the disordered site energy distribution. The black solid lines represent energy levels of NCs in the solid.

In an ideal system of nanocrystals, every NC has the exact size, shape, composition, and surface. These ideal nanocrystals would assemble in an orderly manner to form superlattices. In real systems, however, each NC is slightly different from the next which leads to a distribution of band gap energies.^13^ Since the first colloidal semiconductor synthesis was reported around three decades ago, significant advances have been made for better control on size polydispersity, surface ligand placement and shape uniformity. Even in highly monodisperse NCs, however, energetic disorder is still present.^50^ When charge carriers are formed on a subset of NCs, the energy difference serves as one of the thermodynamic driving forces for charge carriers to explore their surroundings, and preferentially dwell on the NCs with lower site energies (*i.e.* smaller band gap or lower potential of the relevant transport level) (Fig. 3).^51–53^ The energetic disorder therefore has important implications for charge transport, particularly early in the carrier lifetime.

Two commonly used transient optical techniques are transient photoluminescence (PL) and ultrafast transient absorption (TA). Transient photoluminescence tracks the recombination of electron–hole pairs while transient absorption tracks the change in electronic state occupation. The timescales of carrier dynamics routinely probed by these two techniques are also different: while TA usually detects dynamics on the timescale of femtosecond (fs) to nanosecond (ns), PL complements it by providing information on the timescale of sub-nanosecond to microsecond (μs). Kagan and co-workers first demonstrated the dipolar coupling between two neighboring NCs by measuring the time-resolved PL from film samples with mixed size NCs.^51,52^ Since then, the PL quenching from small NCs, in combination with PL enhancement from large NCs, has been used as conclusive evidence of excitonic energy transfer between NCs. However, even in a “single” size NC ensemble, effects of inhomogeneous migration can be observed.

**Fig. 4:**
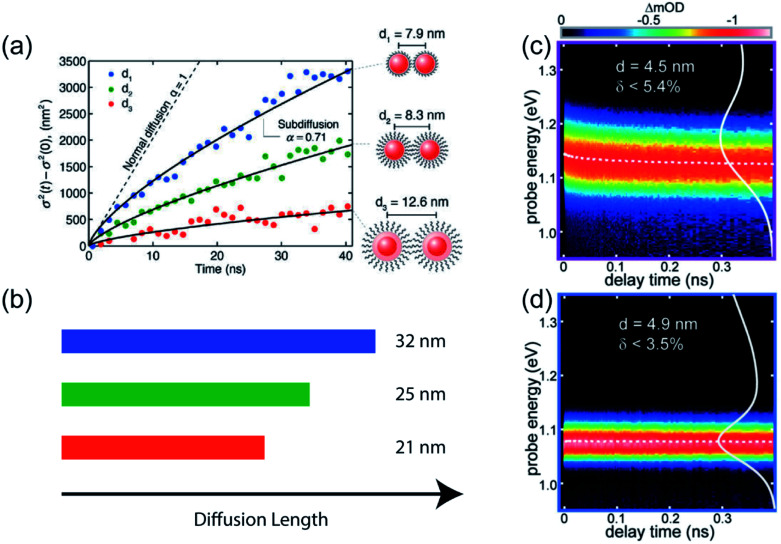
(a) Change in variance, *σ*^2^, of the exciton distribution as a function of time in three different CdSe/CdZnS core/shell NC samples, as measured by transient photoluminescence microscopy. Solid lines are fits to a power law (reprinted with permission from ref. 40. Copyright 2014 American Chemical Society). (b) Extracted exciton diffusion length for the three samples. (c and d) Spectrally-resolved transient absorption in two PbS solid films having different polydispersity (reprinted with permission from ref. 22. Copyright 2017 American Chemical Society).

Transient photoluminescence microscopy revealed that within a “single” size CdSe NC ensemble, exciton diffusion does not follow a true random-walk process; instead excitons migrate energetically downhill, resulting in decreasing diffusivity of the exciton population as time progresses (Fig. 4a and b).^40^ In this type of spatiotemporal measurement, transient photoluminescence is collected as a function of spatial position, allowing direct visualization of charge/exciton diffusion after the laser pulse arrival.^46^ In these CdSe NC solids, the diffusivity of the exciton population was observed to decrease over time. This behavior was attributed to the presence of energetic disorder within the NC solid. The existence of energetic disorder decreases the late-time diffusivity and, macroscopically, the average effect of disorder is to reduce overall exciton transport.^54^ The spatiotemporal measurements were complemented by spectrally-resolved transient PL, highlighting the importance of capturing time-dependent carrier dynamics in understanding the photophysical fate of the carriers following laser excitation.

TA spectroscopy has been utilized similarly to follow how the lowest electronic transition bleach changes as a function of delay times and enabling dynamics to be resolved on a faster timescale than PL-based spectroscopy. Through a comparison between PbSe NC dispersion and solids, it was found that strong electronic coupling of NCs leads to a fast interparticle thermalization of charge carriers, visualized by a spectral diffusion on picosecond (ps) timescale.^55^ Similarly, previous work from our group compared the magnitude and rate of transient red shifts of the band-edge bleach signal across a wide series of NC sizes and batch polydispersities.^22^ We used the rate of red shifting, aided by kinetic Monte Carlo (KMC) simulations, to calculate the carrier hopping rate and quantify homogeneous and inhomogeneous contributions to the ensemble absorption and emission linewidths (Fig. 4c and d). The kinetic Monte Carlo simulations were used to describe the dynamics of independent free charge carriers diffusing within a three-dimensional array of NCs and to generate a microscopic physical interpretation of the experimental data. In the simulation, the behavior of free carriers was assumed to follow the Miller–Abrahams rate equations with the intrinsic hopping rate, *k*′, and the inhomogeneous distribution of site energies, *σ*_inhom_, as the only two fitting parameters. The spatial distribution of NCs was modeled as a BCC lattice, derived from grazing-incidence small-angle X-ray scattering (GISAXS) data. The simulations were able to capture the experimentally observed behavior using realistic physical parameters, validating the microscopic interpretation of nonequilibrium charge carrier dynamics at early times.^22^

TA microscopy has also been used to probe exciton transport in CdSe NC solids, where it was shown that exciton diffusivity is higher in CdSe superlattices than in disordered CdSe NC solids.^42^ Spatiotemporal techniques have evolved beyond PL- and TA-based approaches as well. In a recent demonstration, Ginsberg and co-workers described the use of an optical scheme that leverages interferometric scattering,^49^ which is sensitive to charge, exciton, and heat transport.

In an ideal experiment, temporal, spectral, and spatial information would be collected simultaneously; however, this is often not the case due to practical limitations. Spectrally-resolved transient measurements and spatially-resolved transient measures, therefore, often complement each other, collectively providing a more complete picture of charge carrier dynamics than either approach alone. We note that while spectrally-resolved transient optical spectroscopy techniques provide valuable information about the disordered energy landscape charge carriers sample during their lifetime, these ensemble-averaged approaches *rely* on the existence of disorder to extract microscopic insight.^22,36,56^ Transient microscopy, on the other hand, captures charge carrier transport even in the lack of energy disorder.^45,46^

## Carrier trapping limits device performance

**Fig. 5:**
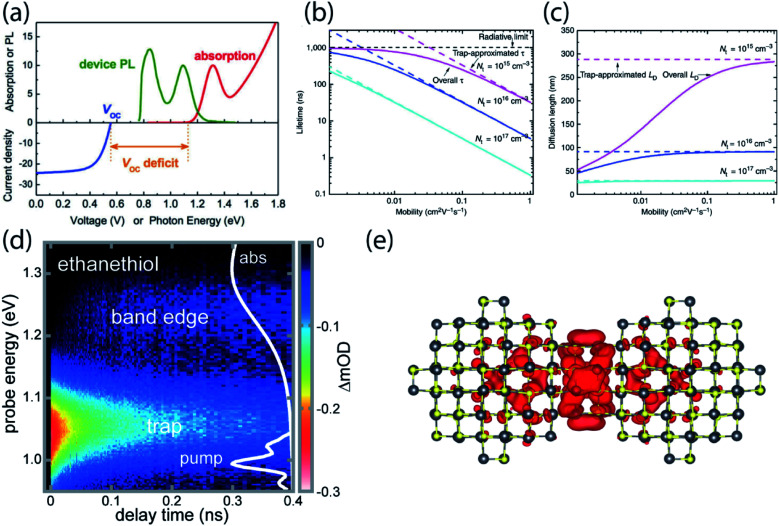
(a) Absorption spectrum, photoluminescence and *V*_OC_ of a PbS NC film used in a solar cell device. *V*_OC_ deficit is usually larger than 500 meV (reprinted with permission from ref. 57. Copyright 2015 American Chemical Society). (b and c) Theoretical lifetime and diffusion length for several mobilities and trap state densities close to experimental values. The dashed lines represent the expected values without consideration of trap states (reprinted with permission from ref. 58 Copyright 2014 Springer Nature). (d) Transient absorption of PbS NC solid treated with ethanethiol following direct photoexcitation of a shallow trap. Carriers are entropically driven uphill in energy to the band edge. (e) The wavefunction of the LUMO of a dimer with 12 atoms in the fused plane (reproduced from ref. 61 with permission by Cell Press).

While the application of nanocrystals in display technologies has achieved commercial success, the use of NCs in photovoltaic cells is lagging behind. For solar cells, PbS quantum dots are a promising candidate due to the near-infrared bandgap and broad tunability for potential multi-junction solar cell fabrication. The certified power conversion efficiency has increased from 3% to over 12% over less than a decade, and device stability has improved drastically to over 1000 hours of operation.^4^ Despite progress in the field, the power conversion efficiency, determined by open-circuit voltage (*V*_OC_), short-circuit current (*J*_SC_) and fill factor, is still much lower than the performance expected for such a low-bandgap semiconductor. Among these three parameters, the open-circuit voltage shows the largest deficit compared to the theoretical expectation. Mathematically, *V*_OC_ is expected to be the same as the semiconductor band gap divided by the electron charge, *E*_g_/*q*, where *E*_g_ is the absorbance onset of the semiconductor, but a deficit of larger than 500 meV is consistently observed (Fig. 5a).^57^

In ideal NC solids, no electronic state exists between the valence band and the conduction band. Real NC solids, however, often have “trap” states that exist between the two band edge states. These states are characterized as “deep” or “shallow” depending on their energetic proximity to either band edge. Trap states can act as recombination centers that directly inhibit charge extraction out of the NC active layer.

The carrier diffusion length is a fundamental materials parameter that affects the design of photovoltaic devices. The carrier diffusion length must be larger than the thickness of active layers in devices for photogenerated charges to be collected efficiently. Both the charge carrier mobility and the carrier lifetime determine the carrier diffusion length, defined frequently as 
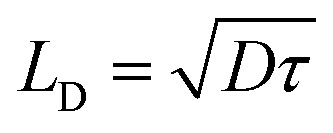
. However, when mobility and lifetime are measured separately in NC solids, it often leads to an overestimate of the diffusion length. In typical NC solids, the minority charge carrier diffusion length is determined not by the mobility of charge carriers, but rather by the average spatial separation of recombination centers (*i.e.* trap states) (Fig. 5b and c).^58^

The origin of these traps has been one of the most debatable topics in the NC community given its importance in NCs' optoelectronic performance. The most commonly cited origin is the surface of the NCs as a very high fraction of atoms in a crystal is on the surface.^59,60^ In addition, the most efficient method to eliminate traps is passivating the surface – either through ligand exchange/treatment or shell growth. For various metal chalcogenide NCs, nonstoichiometry on the surface leads to dangling bonds, which create electron and hole traps. These electronic states originating from the surface are expected to have very little oscillator strength.

Very recently, we presented evidence that epitaxial dimerization of two nanocrystals will form an optically active trap state, and that this source of trap states shares many characteristics with the trap states inferred from electrical device measurements and photoluminescence spectroscopy.^61^ We used ultrafast transient absorption to directly photoexcite the below-gap ground-to-trap transition and subsequently followed the population dynamics in time (Fig. 5d). We found that the absorption cross section and degeneracy of these trap states is similar to larger NCs, and that this behavior could be explained by the presence of atomically aligned nanocrystal dimers, which were observed under high-resolution TEM and calculated from density functional theory (DFT) (Fig. 5e). Using temperature-dependent PL we inferred a trap state density of about 1 in ∼2500 in ethanethiol-terminated PbS NC solids. However, this trap state density was about 1000 times higher than in the native oleate-capped NC solids. It is therefore suggested that while solid-state ligand exchange passivates the surface, it strips off ligands, creating opportunities for neighboring NCs to fuse. Solution-phase ligand exchange might therefore be more preferable for surface passivation.^37,62–64^ However, there may be additional non-optically-active trap states that affect electrical measurements and other facts that contribute to the open-circuit voltage deficit in PbS NC photovoltaics.

## Carrier de-trapping can be understood by equilibrium thermodynamics

We identified two different kinetic mechanisms of carrier detrapping in PbS NC solids: thermally-activated hopping and Auger-assisted charge transfer. Following direct photoexcitation into the trap state manifold, the trap state signal decayed (over ∼20–500 ps timescale) while a signal corresponding to occupation of the band edge grew in. This was a surprising result as the trap state was ∼180 meV lower in energy than the band edge – considerably deeper than the available thermal energy at room temperature, ∼25 meV. Careful analysis of fluence- and temperature-dependent de-trapping kinetics revealed two distinct mechanisms: thermally-activated de-trapping and Auger-assistant de-trapping.

To understand the counterintuitive observation of ∼180 meV uphill migration of the carrier population at room temperature, entropy needs to be considered. After the initial pulsed laser excitation, charge carriers quickly establish a Boltzmann distribution over the electronic density of states within the NC array. The ratio of charge carrier occupation in the band edge manifold to the trap state manifold depends not only on the energy difference between these levels and the sample temperature, but also on the relative degeneracy of these levels,4
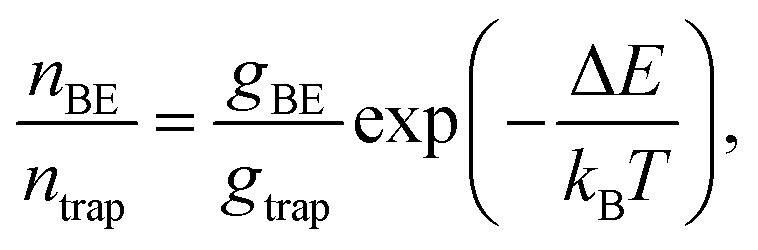
where *g*_BE_ and *g*_trap_ are the degeneracy of the band edge manifold (here the term “manifold” denotes the collection of all states in the NC solids having similar physical origin) and the trap state manifold, respectively, Δ*E* is the energy difference between these two manifolds, *k*_B_ is the Boltzmann constant and *T* is temperature. Even though occupation of the trap state is energetically preferred at room temperature, the entropy gained by occupation of the band-edge is enough to shift the equilibrium expectation value close to the band edge.

We emphasize that it is indeed possible for the carrier population to reach equilibrium between the band edge and trap state manifolds during the average carrier lifetime. KMC simulations of nonequilibrium charge carrier dynamics in the strong electronic coupling regime have validated the microscopic interpretation of our transient absorption data.^22^ A thermodynamic equilibrium is established within a few nanoseconds, consistent with the saturation of band-edge and trap state occupancies observed *via* TA (Fig. 5d). In fact, the microscopic transition rates between trap states and band edge states can be very fast due to efficient multi-phonon assisted transitions in lead salt NCs.^65^

## Unique dynamics arise from multicarrier interactions

**Fig. 6:**
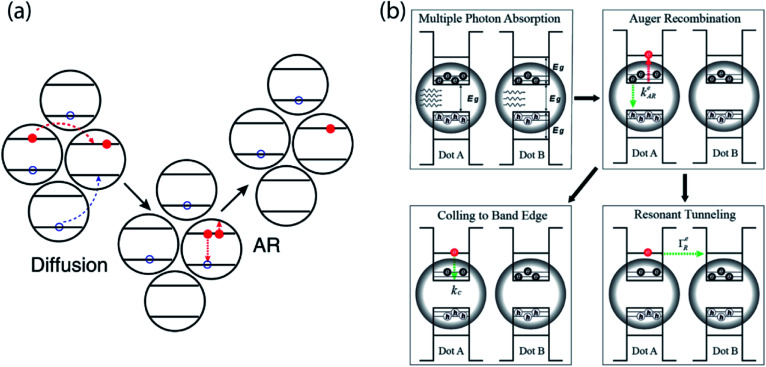
(a) Cartoon showing that disorder enhances Auger recombination of charge carriers at low-energy site (reprinted with permission from ref. 43 Copyright 2013 Springer Nature). (b) Schematics revealing the possible mechanism of hot carrier resonant tunneling (reprinted with permission from ref. 69. Copyright 2019 American Chemical Society).

In NC devices, the flow of charge carriers is often influenced by interaction with other charge carriers, especially under high excitation conditions. In particular, the disordered energy landscape of NC solids drives photogenerated carriers to low-energy sites, making them the hot spots of the solids where multi-carrier interaction can occur.^43^ The accumulation of charge carriers in these sites drastically increases the likelihood of Auger processes despite overall low excitation density. Gao *et al.* used time-resolved microwave conductance and transient absorption in conjunction with KMC modeling to show that even at fluences as low as 1 excitation per ∼1000 NCs, disorder-enhanced Auger recombination can occur. The high mobility of charge carriers can further speed up this decay pathway, leading to faster carrier annihilation (Fig. 6a).

We recently observed that multi-carrier occupation of single trap sites can enable Auger-mediated de-trapping processes. In the case of PbS NC solids, the thermally-activated de-trapping pathway described in the previous section was frozen out at low temperature; however, even at 80 K, carriers could still escape these shallow traps through an Auger-assisted pathway. In the Auger-assisted picture, two charge carriers occupy the same trap site. One carrier returns to the ground state, giving its excess energy to the second carrier; this transiently hot carrier can then tunnel quickly to a neighboring NC.

Another multi-carrier interaction – carrier multiplication (CM) – has long excited the nanocrystal research community because of the possibility of leveraging this approach to surpass traditional limits for solar power conversion efficiency.^66,67^ Carrier multiplication is a Coulomb-mediated many-body interaction in which a single high-energy electron–hole pair is converted to two lower-energy electron–hole pairs in an energy-conserving process. Klimov and co-workers used picosecond transient photocurrent to quantify extracted multi-carrier yields in NC solids exhibiting carrier multiplication.^68^ Importantly, this approach revealed that Auger recombination competes with the charge extraction process, limiting overall yields. Although carrier multiplication primarily occurs within a single NC, clearly these single-NC dynamics must be matched to mesoscale charge transport dynamics for efficient multicarrier extraction. In a recent study, Alivisatos & Rabani and co-workers used picosecond transient photocurrent to show that, following photoexcitation with a femtosecond laser pulse, Auger recombination in the same NC can promote fast resonant tunneling through hot electron states between neighboring PbSe NCs (Fig. 6b).^69^

## Looking forward: anisotropic nanocrystals and halide perovskites

Recent advances in nanocrystal materials, including anisotropic nanocrystals, such as nanorods and nanoplatelets, and halide perovskite nanocrystals, present new opportunities for exploring mesoscale dynamics in NC arrays. Different from isotropic nanocrystals, nanoplatelets and nanorods are only quantum confined in one dimension and two dimensions, respectively.^70,71^ This intrinsic shape anisotropy leads directly to anisotropy in electronic and optical properties.

Directional charge carrier transport in anisotropic NCs can minimize the path necessary for carriers to be extracted out of the active layer of the devices.^72^ For example, in photovoltaics, photogenerated charge must migrate through the NC active layer to electrode interfaces for charge extraction. In active layers composed of spherical or cubic nanocrystals, carrier transport is isotropic in three dimensions, despite a one-dimensional device structure. Layers of aligned anisotropic NCs could reduce the number of hops required to reach a collecting electrode, thus minimizing the chance trapping or annihilation. Time-resolved microscopy is ideally suited for resolving such anisotropic transport phenomena.^73^

Optical anisotropy can aid in improving the external quantum efficiencies of light-emitting diodes (LEDs) through light outcoupling engineering.^74,75^ When the emissive nanoparticles are orientated with their transition dipole parallel to the surface plane, the emission becomes directional, enhancing the device efficiency. This optical anisotropy also manifests in anisotropic rates dipole-mediated energy transfer.^76^ Excitonic energy transfer between parallel 2D NCs can be exceptionally fast. In well oriented lead halide perovskite quantum wells, excitonic energy transfer between the two nanoplatelets occurs on a timescale of 100's of femtoseconds.^77^ Energy transfer on picosecond timescale has also been observed in CdSe nanoplatelets.^78^ As these excitonic energy transfer processes are understood to be dipole mediated, it is unclear whether similar benefits may be realized for charge transport in these materials.

Over the past five years, perovskite nanocrystals have emerged as an extremely promising nanomaterial platform.^79–84^ As the bulk perovskite-based photovoltaic efficiency approaches that of single crystal silicon, stability remains a critical issue.^85^ Low-dimensional perovskites have been proposed as a potential solution to this problem due to the presence of hydrophobic surface ligands.^86^ These surfactants serve as barriers for water molecules to penetrate into the perovskite lattice; in addition, these surface molecules can help stabilize specific perovskite phases.^87^ Solar cells based on CsPbI_3_ NCs or CsPbI_3_/FAPbI_3_ NC hybrids have been demonstrated with efficiencies that surpass PbS NC photovoltaics.^88,89^ High device efficiency in perovskite nanocrystal solar cells has been attributed to the enhanced mobility of charge carriers in CsPbI_3_ NC arrays treated with FAI (formamidinium iodide), measured by time-resolved terahertz spectroscopy.^88^ These initial studies suggest an important role for time-resolved spectroscopy in elucidating microscopic insight in perovskite NC solids.

Additionally, perovskite nanocrystals exhibit intriguing excitonic behavior that might enable novel transport phenomena. One such observation is that of mesoscopically extended coherent states when the colloidal perovskite nanocrystals assemble into superlattices.^82^ As described in previous sections, the dominant carrier transport mechanism in inhomogeneously broadened NC solids is site-to-site hopping. However, coherent transport mechanisms might be possible in NC superlattices with sufficiently low energetic disorder and sufficiently strong nearest-neighbor coupling. Using steady-state and transient photoluminescence, as well as correlation measurements, Raino *et al.* characterized superfluorescence from long-range-ordered CsPbBr_3_ NCs that was absent in glassy films. Superfluorescence occurs when the initially uncorrelated transition dipoles on different NCs establish coherence and collectively oscillate in phase. Theoretically, coherent or partially coherent exciton transport can dramatically increase exciton diffusivity.^90^ This is potentially useful in solar energy harvesting and photon upconversion devices where maximizing the exciton diffusion length is necessary.^90^

## Closing remarks

As these examples demonstrate, a time-domain view of charge carriers in nanocrystal solids offers a compelling complement to traditional understanding of equilibrium transport phenomena in disordered semiconductors. Transient measurements are necessary to elucidate the time-dependent charge carrier motions. With powerful tools developed in recent years, our understanding of carrier transport in NC solids has been reexamined and refined. Emerging semiconductor nanostructures, including anisotropic nanocrystals and perovskite nanocrystals, have opened up new doors for further exploration of new photophysical phenomena.

## Conflicts of interest

There are no conflicts to declare.
